# A case report of Posterior reversible encephalopathy syndrome with spinal cord involvement (PRES-SCI) as an atypical presentation of PRES in children: A case report and review of the literature

**DOI:** 10.22037/ijcn.v16i1.32170

**Published:** 2022-03-14

**Authors:** Javad AKHONDIAN, Farah ASHRAFZADEH, Farrokh SEILANIAN TOOSI, Mahdi BEHNAM, Mehran BEIRAGHI TOOSI, Shima IMANNEZHAD, Mohammad Reza AKHOUNDIAN, Narges HASHEMI

**Affiliations:** 1 Department of Pediatrics , School of Medicine, Mashhad University of Medical Sciences, Mashhad, Iran.; 2 Department of Radiology, School of Medicine, Mashhad University of Medical Sciences, Mashhad, Iran.; 3 General physician, School of Medicine, Mashhad University of Medical Sciences, Mashhad, Iran.

**Keywords:** Posterior reversible encephalopathy syndrome, Spinal cord, Hypertension, Pediatrics

## Abstract

Posterior reversible encephalopathy syndrome (PRES) has a broad spectrum of clinical presentations and radiological features. Diagnosis of PRES is established based on reversible clinical manifestations and sequential neuroimaging findings. Atypical MRI features include hemorrhage, restricted diffusion or contrast enhancement of lesions, and involvement of the temporal and frontal lobes, brainstem, basal ganglia, corpus callosum, cerebellum, and spine. Atypical PRES, with or without spinal cord involvement, is a rare presentation, especially in children. Until 2020, only five cases of PRES with spinal cord involvement (PRES-SCI) were reported in the pediatric population.

**Case Report:**

Here, we present the youngest diagnosed case of PRES-SCI so far. According to the literature, all six cases of PRES-SCI showed high signal intensities on T2-weighted images of the brainstem and cervical cord, which had completely resolved in the follow-up MRI of the brain and spinal cord. All six patients had hypertension due to renal disease, except one girl with chemotherapy-induced hypertension. Headache, altered mental status, seizure, and visual impairment were the most common symptoms, respectively. Facial palsy was a remarkable warning sign in some patients before hospitalization.

Although PRES-SCI is rare in children, since it is a reversible condition, prompt diagnosis and management can positively affect its prognosis.

## Introduction

Posterior reversible encephalopathy syndrome (PRES) is a life-threatening disorder, characterized by altered mental status, seizure, and edema, predominantly in the parietal and occipital regions. It is often reversible if managed promptly within days or weeks. It is associated with several clinical conditions, including hypertension, vasculitis, organ transplantation, renal disease, and autoimmune or malignant disorders (1, 2). Other reported etiologies include eclampsia, intravenous immunoglobulin administration, thrombotic thrombocytopenic purpura, hemolytic uremic syndrome, polyarteritis nodosa, sickle cell disease, and systemic lupus erythematosus (1-3).

The diagnosis of PRES is established based on the presence of an underlying disease, reversible clinical manifestations, and sequential neuroimaging findings. Typical neuroimaging findings include hypersignal subcortical white matter involvement on T2-weighted images, predominantly in the parietal and occipital regions (1, 4, 5). Atypical magnetic resonance imaging (MRI) features include hemorrhage, restricted diffusion or contrast enhancement of lesions, and involvement of the temporal and frontal lobes, brainstem, basal ganglia, corpus callosum, cerebellum, and spine (6, 7).

Herein, we present a case of PRES with spinal cord involvement (PRES-SCI) and describe other cases reported in the literature, with or without other atypical presentations of PRES.

## Case Report

A four-year-old girl presented with asymmetrical laughing and inability to close her right eye. In her clinical examination, right peripheral seventh nerve palsy was observed. Other neurological examinations were normal. She was conscious with no other complaints. She was discharged with prednisolone and acyclovir and advised to return for a follow-up within three days. However, after six days, she was admitted to the emergency department with altered consciousness and status epilepticus. Her blood pressure was 180/110 mmHg in the emergency room.

**Figure 1:**
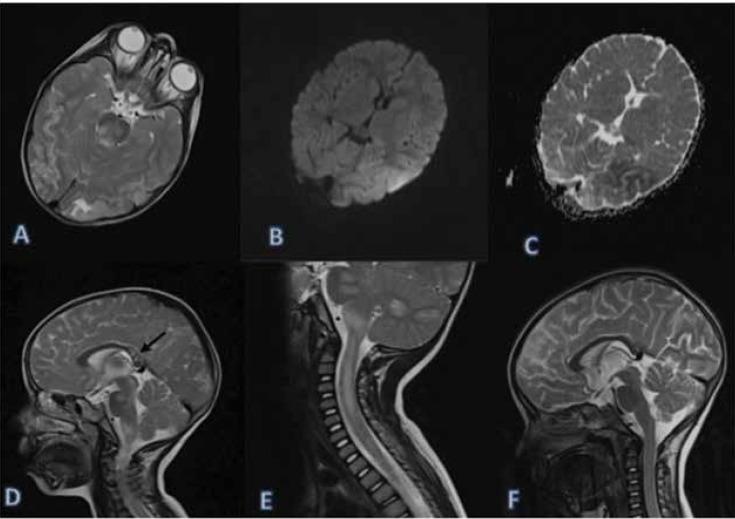
Axial brain MRI shows high signal intensities in the parietal and occipital regions, as well as the brainstem on T2-weighted images (A). Restricted diffusion on DWI and ADC maps (B, C). Sagittal T2-weighted images show abnormal signal intensities in the splenium of the corpus callosum (black arrow) and the brainstem (D). Abnormal signal intensities in the brainstem, cerebellum, and cervical cord (E). Normal follow-up MRI of the brain and spinal cord after one month (F)

Brain MRI was performed on the first day of hospitalization. Abnormal signal intensities were observed in the parietal and occipital lobes with restricted diffusion on the left side (Figures 1A, 1B & 1C). The splenium of the corpus callosum, cerebellum, brainstem, and cervical cord were involved severely (Figure 1D & 1E). According to the clinical and radiological findings, a diagnosis of acute disseminated encephalomyelitis (ADEM) or atypical PRES was suspected.

Seizure was managed with phenytoin and phenobarbital. Hypertension was also controlled with labetalol, Lasix, hydralazine, and captopril in one week. Etiological findings revealed high plasma renin activity and high aldosterone levels. The results of CT angiography indicated stenosis in the right renal artery. Accordingly, a diagnosis of secondary hyperaldosteronism was made as the cause of hypertension.

In further examinations, there was no sign of vasculitis. Also, in the one-month follow-up, there were no signal abnormalities in the brain and spinal cord MRI (Figure 1F). The patient experienced no neurological insults, except right peripheral facial nerve palsy. Considering the reversibility of the condition, an atypical variant of PRES was the most likely diagnosis. She showed severe hypertension upon admission due to secondary hyperaldosteronism, which was possibly caused by prednisolone consumption.

## Discussion

PRES-SCI was first described by de Havenon A et al. (8). Spinal cord involvement is the rarest radiological finding of PRES. All cases of PRES-SCI are complicated by brainstem lesions, and there is no report of isolated PRES-SCI in adults or children. Cerebrovascular autoregulation failure due to hypertension leads to vasogenic edema, which is reversible if promptly treated. Diffuse restriction on ADC maps suggests cytotoxic edema and potentially irreversible ischemia. One hypothesis is that edema in the spinal cord may be relative to the vertebrobasilar artery supply disturbances. However, the rarity of this condition is due to dense sympathetic innervations of the spinal cord. Considering the low density of sympathetic innervations in the vertebrobasilar system, vasogenic edema commonly occurs in the posterior brain zones (8, 9). Nevertheless, this mechanism cannot be justified in a quarter of patients with normal blood pressure (10). Another hypothesis suggests vasculopathy due to endothelial dysfunction (10, 11).

PRES-SCI is assumed to be a subtype of PRES, which is commonly associated with brainstem involvement. Except for our case, five other pediatric cases of PRES-SCI have been described in the literature since 2000 (8, 12-15). Overall, 80% of these cases were female, despite a male predilection in adults (16). Headache and altered mental status were the most common manifestations of typical PRES (16). Severe hypertension due to renal impairment has been reported in previous studies, except for one patient with acute lymphocytic leukemia and subsequent chemotherapy (12). All cases reported in the literature showed high signal intensities on T-weighted and fluid-attenuated inversion recovery (FLAIR) images of the brainstem and spinal cord, which completely resolved in the follow-up MRI. The patients’ MRI findings of the brain and spine abnormalities, demographic factors, and underlying disorders are presented in Table 1.

The first presentation of our patient was unilateral peripheral facial palsy six days before experiencing complications. In the literature, there is a report of a nine-year-old girl with two attacks of facial palsy during one year before diagnosis (13). Considering the reversible clinical and radiological features, the diagnosis of atypical PRES was established. Our patient showed severe hypertension upon admission due to renal artery stenosis, which was possibly caused by prednisolone consumption. Overall, in the differential diagnosis of this condition, cerebral venous thrombosis, encephalitis, acute disseminated encephalomyelitis, central pontine myelinolysis, and myelitis should be considered (3, 15).

Before and after recovery, comparison of ADC maps with DWI sequences of MRI scans plays an important role in distinguishing reversible vasogenic edema from other possible conditions. Prompt diagnosis and early management of this condition can decrease the rate of complications and improve the outcomes in children. Since facial nerve palsy can be the first manifestation of PRES-SCI patients without any other complaints, accurate clinical examination and blood pressure measurement are critical.

**Table 1:** Underlying diseases, demographic characteristics, and radiological findings of five cases reported in the literature

case	Age/ gender	Blood pressure	Underlying disorder	MRI finding/typical	MRI finding/Atypical	Neurologic out come
1	4/F	118/110	Unilateral renal artery stenosis	Occipital parietal	Cervical cord Brain stem, Corpus callosum Temporal, frontal cerebellum	recovery
2	7/M	190/100	Reflux Nephropathy	parietal	Cervical cord Brain stem Basal ganglia	recovery
3	10/F	140/105	chemotherapy	-	Cervical cord Brain stem Cerebellum	recovery
4	9/F	220/110	Reflux Nephropathy	-	Spinal cord Brain stem	recovery
5	14/F	145/85	Renal artery stenosis	-	Cervical cord Brain stem Cerebellum	recovery
6	14/F	2	Reflux Nephropathy	Parietal	Spinal cord Brain stem	recovery

## Author’s Contribution

Narges Hashemi conceptualized and designed the report, wrote the manuscript, and contributed to all stages of this case report. Javad Akhondian, Farah Ashrafzadeh, and Mahdi Behnam were the attending physicians for the patient. Farrokh Seilanian Toosi reported the imaging findings. Shima Imannezhad was responsible for data collection. Mohammad Reza Akhoundian revised the manuscript and contributed to the drafting of the manuscript. All authors read and approved the final manuscript. Narges Hashemi is the guarantor of this study.

## Conflict of Interest

The authors declare no conflicts of interest.
